# *EDA* Mutations Causing X-Linked Recessive Oligodontia with Variable Expression

**DOI:** 10.3390/genes16010012

**Published:** 2024-12-26

**Authors:** Ye Ji Lee, Youn Jung Kim, Wonseon Chae, Seon Hee Kim, Jung-Wook Kim

**Affiliations:** 1 Department of Pediatric Dentistry & DRI, School of Dentistry, Seoul National University, Seoul 03080, Republic of Korea; yeaji24@snu.ac.kr (Y.J.L.); ykim71@snu.ac.kr (Y.J.K.); cws50055752@gmail.com (W.C.); shsunnyten19@gmail.com (S.H.K.); 2 Department of Molecular Genetics & DRI, School of Dentistry, Seoul National University, Seoul 03080, Republic of Korea

**Keywords:** *EDA*, missense mutation, oligodontia, protein model analysis, X-linked

## Abstract

Background/Objectives: The ectodysplasin A (*EDA*) gene, a member of the tumor necrosis factor ligand superfamily, is involved in the early epithelial–mesenchymal interaction that regulates ectoderm-derived appendage formation. Numerous studies have shown that mutations in the *EDA* gene can cause X-linked ectodermal dysplasia (ED) and non-syndromic oligodontia (NSO). Accordingly, this study aimed to identify the causative genetic mutations of the *EDA* gene. Methods: We investigated *EDA* gene mutations in two X-linked oligodontia families using candidate gene sequencing and whole-exome sequencing, with a single proband identified and studied for each family. The first family included a patient with NSO, while the second family had a patient exhibiting variable expression of ED. Results: Mutational analysis identified two missense mutations in the *EDA* gene (NM_001399.5): one novel mutation, c.787A>C p.(Lys263Gln), in family 2; and one previously reported mutation, c.457C>T p.(Arg153Cys), in family 1. All mutated residues are evolutionarily highly conserved amino acids. The p.(Arg153Cys) mutation would destroy the furin recognition site and affect the cleavage of EDA. The p.(Lys263Gln) mutation in a TNF homology domain would interfere with the binding of the EDA receptor. The p.(Lys263Gln) mutation was associated with NSO, while the other mutation demonstrated ED. Conclusions: This study helps to better understand the nature of EDA-related ED and NSO and their pathogenesis, and it expands the mutational spectrum of *EDA* mutations.

## 1. Introduction

Tooth agenesis represents one of the most common craniofacial anomalies in humans and is estimated to affect approximately 2–9% of the population [1]. It can be categorized into hypodontia, oligodontia, and anodontia, depending on the number of missing teeth, excluding the third molars [2]. Oligodontia is defined as the congenital absence of six or more teeth. Anodontia, an extremely rare condition, refers to the complete absence of all teeth. It also can be classified into non-syndromic and syndromic tooth agenesis according to the accompanying symptoms [3].

Any disturbance in tooth development involving a series of inductive interactions between the epithelium and underlying mesenchyme may result in tooth agenesis or other dental defects [4]. To date, a large number of mutations have been reported to be associated with this condition [5,6]. Specifically, mutations in the *MSX1* (OMIM *142983), *PAX9* (OMIM *167416), *AXIN2* (OMIM *604025), *LRP6* (OMIM *603507), *WNT10A* (OMIM *606268), and *EDA* (OMIM *300451) genes have been shown to cause non-syndromic and/or syndromic tooth agenesis [7,8,9]. Among these genes, mutations in *EDA* are typically associated with both syndromic tooth agenesis, particularly ectodermal dysplasia (ED), and non-syndromic oligodontia (NSO) [10,11]. X-linked ED represents a hereditary condition characterized by congenital defects of the epithelial appendages, including the hair, nails, teeth, and sweat glands, and is inherited as an X-linked recessive trait. Affected subjects usually present with hypohidrosis, hypodontia, sparse hair, abnormal development of nails, and characteristic face [12]. Recently, several other current studies have identified *EDA* mutations and characterized genotype–phenotype relationships [13,14,15,16,17].

The *EDA* gene is localized on Xq12-q13 [18] and encodes a trimeric transmembrane protein that constitutes a member of the tumor necrosis factor (TNF) ligand superfamily [19]. EDA is involved in the early epithelial–mesenchymal interaction that regulates ectoderm-derived appendage formation, such as the teeth, hair, and eccrine and mammary glands [20]. This protein comprises a small N-terminal intracellular domain, a transmembrane domain, a larger C-terminal extracellular domain that contains a furin recognition site, a collagen-like domain, and a TNF homology domain. EDA has to be released from the full-length precursor protein by proteolytic processing at the furin cleavage site as a soluble homotrimer protein consisting of the collagen-like and TNF homology domains to bind to its receptor [21]. As a result of proteolysis, the soluble EDA homotrimer can initiate the EDA signaling pathway [22]. This pathway has three major components consisting of the TNF ligand–receptor–adaptor family proteins [23]. Specifically, the TNF domain of EDA directly binds to the extracellular region of the death domain-containing EDA receptor (EDAR). The EDAR (OMIM *604095), in turn, interacts with an adaptor molecule, the EDAR-associated death domain (EDARADD, OMIM *606603), to activate the NFκB intracellular signaling pathway [24]. With further involvement of TRAF6, TAK1, and TAB2, the NFκB essential modulator (NEMO)-IκB-NFκB signaling cascade is activated [25]. Consequently, NFκB translocates into the nucleus, stimulating the transcription of many genes that are necessary to initiate, form, and differentiate skin appendages [26].

This study investigated *EDA* gene mutations in two oligodontia families with a single proband identified and studied for each family—one family included a patient with NSO, while the other included a patient with ED—using *EDA* gene sequencing and whole-exome sequencing. Western blotting and 3D protein modeling were used to test and predict potential functional consequences. As a result, we hypothesized that these *EDA* mutations are responsible for tooth agenesis in the affected subjects, with variable expression.

## 2. Materials and Methods

### 2.1. Enrollment of Human Subjects

The study protocol and patient consent were independently reviewed and approved by the Institution Review Board at Seoul National University Dental Hospital (IRB File Number: CRI05003G). Written informed consent was obtained from all participants. Clinical examinations were performed, and panoramic radiographs were taken. Pedigrees were drawn, and family histories were taken. Genomic DNA was extracted from peripheral blood or saliva samples using the NucleoSpin Blood L kit (Macherey-Nagel GmbH & Co., Düren, Germany). The purity and concentration of the isolated DNA were quantitated by spectrophotometry and measured by the OD_260_/OD_280_ ratio.

### 2.2. Candidate Gene Sequencing

The entire coding region and exon–intron boundaries of the *EDA* gene of each proband were amplified by polymerase chain reaction (PCR) using specific primers [3] and the HiPi DNA polymerase premix (Elpis-Biotech, Daejeon, Republic of Korea). PCR products were purified using a PCR Purification Kit according to the manufacturer’s instructions (Elpis-Biotech). DNA sequencing was performed at a DNA sequencing center (Macrogen, Seoul, Republic of Korea). Segregation of the identified mutations within the families was also confirmed with Sanger sequencing.

### 2.3. EDA cDNA Cloning and Mutagenesis

EDA cDNA sequences tagged with FLAG at the C-terminus were amplified from a vector with EDA cDNA cloned in the pJFT7_nHalo_DC(r4) vector (DNASU Plasmid Repository, Tempe, AZ, USA) as a template and cloned into the TopBlunt vector (Enzynomics, Daejeon, Republic of Korea) using primers (forward primer: 5′-AAGCTTACGCGATCGCCACAAGTT-3′; reverse primers: 5′-TCTAGACTACTTGTCGTCATCGTCTTTGTAGTCGGATGCAGGGGCTTCAC-3′). Then, the fragment was double-digested with HindIII and XbaI restriction endonucleases and subcloned into the pcDNA3.1(+) mammalian expression vector. PCR mutagenesis was performed to introduce identified mutations using the following primers (Table 1).

### 2.4. Cell Culture and Transient Transfection

HEK293 cells were transiently transfected with wild-type and mutant plasmids (1 μg each) using Genjet^TM^ Ver. II (Signagen, Frederick, MD, USA) as a transfection reagent. After 36 h post-transfection, the cells and the culture media were harvested. The harvested cells were lysed with 1× cell lysis buffer. The culture media were concentrated with Amicon ultra-4 centrifugal filter units (Millipore, Bedford, MA, USA).

### 2.5. Western Blot

The total protein extracted from the transfected HEK293 cells was quantified and mixed with 5× SDS loading buffer. Protein samples were subjected to 11% SDS-PAGE gel. After gel electrophoresis, the gel was transferred to a PVDF membrane. A primary antibody for EDA (rabbit polyclonal anti-EDA antibody, 25892-1-ap, Proteintech, Rosemont, IL, USA) and a FLAG antibody (mouse monoclonal anti-FLAG M2 antibody, F1804, Sigma-Aldrich, MO, USA) were used at a titer of 1:10,000 and incubated at 4 °C overnight. Secondary antibodies of goat anti-mouse (#G21040, Thermo, Waltham, MA, USA) and goat anti-rabbit (#31460, Thermo) conjugated with HRP were used at a titer of 1:10,000. After using ECL solution (Elpis-Biotech), the membranes were developed in a dark room with X-ray film (Agfa, Elmwood, NJ, USA).

### 2.6. Protein Structure Analysis

Three-dimensional (3D) structural modeling of the EDA protein was performed with the PyMOL software (PyMOL Molecular Graphics System, Version 3.0 Schrödinger, LLC, DeLano Scientific, Palo Alto, CA, USA; http://www.pymol.org/, accessed on 20 June 2024). We used the crystal structure of EDA-A1, Protein Data Bank (PDB: https://www.rcsb.org/, accessed on 20 June 2024) coordinates 1RJ7 and 7X9G [27,28]. AlphaFold was used to predict a 3D structure, and the predicted structure was used for image generation with the PyMOL software [29].

### 2.7. In Silico Prediction of the Mutational Effect

Three widely used in silico prediction programs were used: PolyPhen-2 (http://genetics.bwh.harvard.edu/pph2/, accessed on 13 June 2024) [30], Mutation Taster (https://www.mutationtaster.org/, accessed on 13 June 2024) [31], and CADD score (version 1.7, https://cadd.gs.washington.edu/, accessed on 13 June 2024) [32].

## 3. Results

### 3.1. Family 1

**Figure 1:**
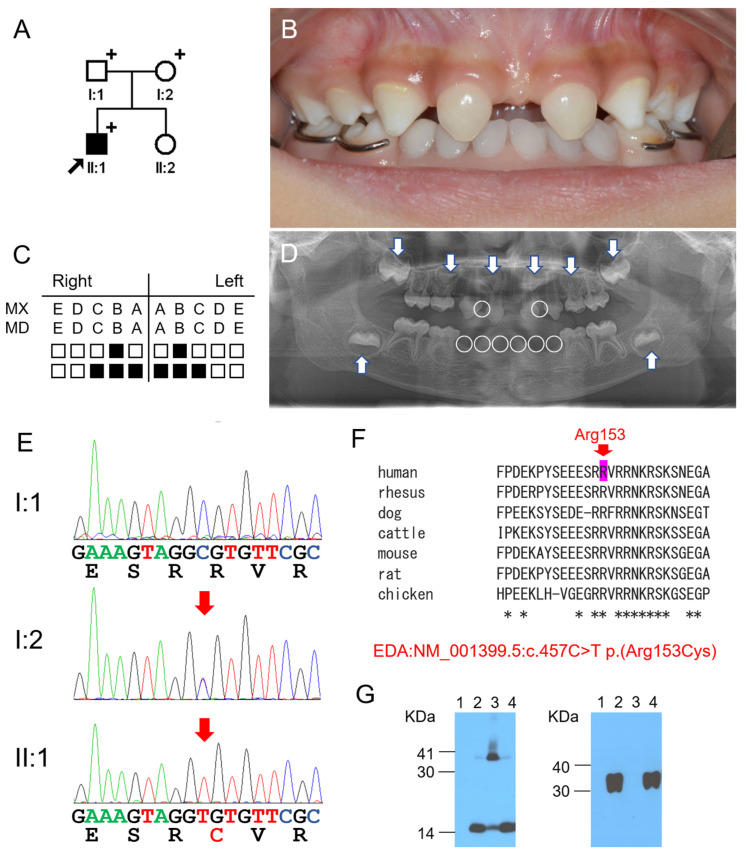
Pedigree, clinical photo, panoramic radiograph, chromatograms, homologene alignment, and Western blot of family 1: (**A**) Pedigree of family 1. A black-filled symbol indicates the affected individual, and the black arrow indicates the proband. Plus signs above the symbols indicate participating individuals. (**B**) Clinical photo of the proband at age 4 years and 3 months. Sharp incisal tips of the deciduous maxillary central incisors were trimmed slightly, and a mandibular removable space maintainer was in place. (**C**) Summary chart of the missing teeth of the proband. She was missing 8 deciduous teeth (the black-filled symbols represent missing teeth). (**D**) Panoramic radiograph of the proband at age 4 years and 1 month. Missing deciduous teeth are indicated with white circles, and identifiable permanent teeth are indicated with white arrows. (**E**) Sequencing chromatograms of the participating individuals of family 1. Nucleotide and amino acid sequences are shown under the chromatograms. The nucleotide affected by the mutation is underlined and indicated with a red arrow (NM_001399.5: c.457C>T p.(Arg153Cys)). Individual identifications are indicated on the left side of each chromatogram. The changed amino acid is shown in red. (**F**) Homologene alignment. The arginine at the 153 codon position is completely conserved among vertebrates and indicated with a red arrow. The asterisk under the alignment indicates the conserved amino acid position (human: NP_001390.1, rhesus: XP_002806316.1, dog: NP_001014770.1, cattle: NP_001075212.1, mouse: NP_034229.1, rat: XP_228582.5, chicken: XP_003641179.1). (**G**) Western blot of cell lysate (left) and culture medium (right). Marker locations and sizes are shown on the left side of the images. The p.(Arg153Cys) mutant (lane 3) was mostly detected in the cell lysate and was not detected in the culture medium (lane 1: empty vector, lane 2: wild-type, lane 3: p.(Arg153Cys), lane 4: p.(Lys263Gln)).

The proband of family 1 was a four-year-old boy who had a severe phenotype (Figure 1). Eight deciduous teeth and up to twenty permanent teeth were missing (Table 2). His maxillary central incisors were characteristically conical-shaped. Clinical examination revealed that the eyebrows were slightly blurred but the hair slightly less so. The patient was found to have fewer sweat glands, and his parents said that he was vulnerable to heat and had difficulty sweating. Based on these characteristics, we concluded that he presented the features of X-linked ED. Other immediate family members, including his sister, had no dental or systemic abnormalities.

Mutational analysis revealed that a recurrent missense mutation (NM_001399.5: c.457C>T p.(Arg153Cys)) was found in exon 2 of the *EDA* gene. The mother was healthy, without any teeth missing. Still, it turned out that the mother also carried the same mutation in heterozygous form. Multiple sequence alignment of the EDA sequence across multiple species was performed using the program ClustalW (https://toolkit.tuebingen.mpg.de/clustalw/, accessed on 20 January 2024). The arginine at the 153 codon position was found to be highly conserved among sequences, including human (NP_001390.1), rhesus monkey (XP_001082424.1), dog (NP_001014770.1), cattle (NP_001075212.1), house mouse (NP_001171408.1), Norway rat (XP_006257173.1), and chicken (XP_003641179.1). In silico prediction values were pathogenic (Table 3). This mutation was previously reported and listed in the dbSNP database (rs397516662). It has been shown to affect furin recognition, which is necessary for proteolytic cleavage. Therefore, the secreted form of the EDA homotrimer is greatly reduced or absent. In this study, we were unable to detect the secreted form of the mutant p.(Arg153Cys) protein in the culture media, whereas secreted forms were observed at similar levels for other mutations and the wild-type EDA.

### 3.2. Family 2

**Figure 2:**
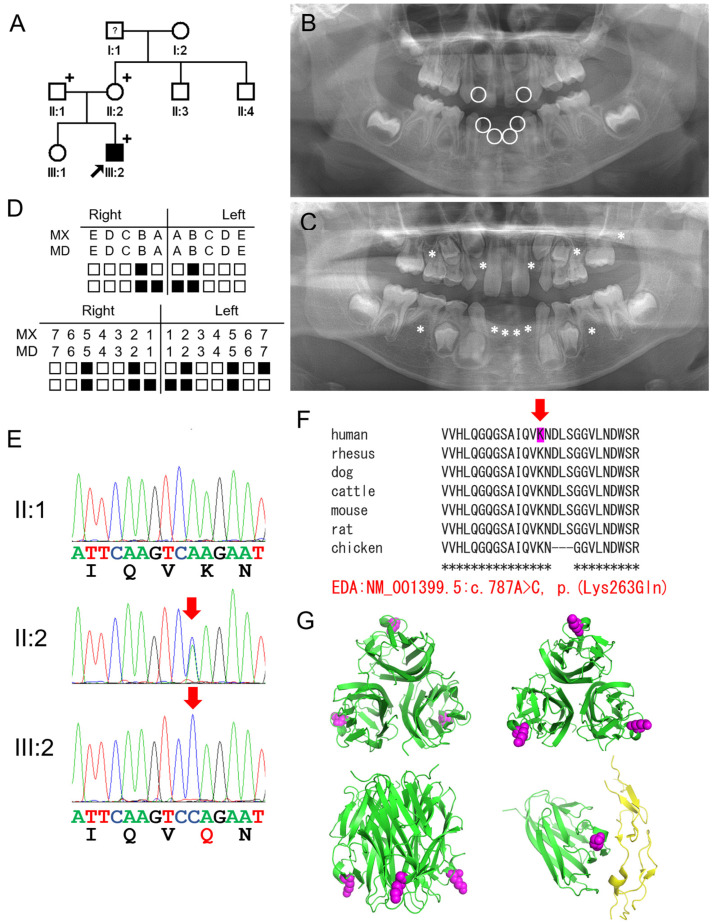
Pedigree, panoramic radiographs, chromatograms, homologene alignment, and 3D protein modeling of family 2: (**A**) Pedigree of family 2. The black-filled symbol indicates the affected individual, and the black arrow indicates the proband. Plus signs above the symbols indicate the participating individuals. (**B**) Panoramic radiograph of the proband at age 3 years and 2 months. Missing deciduous teeth are indicated with white circles. (**C**) Panoramic radiograph of the proband at age 9 years and 8 months. Missing permanent teeth are indicated with asterisks. (**D**) Summary chart of the missing teeth of the proband. He was missing 6 deciduous teeth and 11 permanent teeth (black-filled symbols represent missing teeth). (**E**) Sequencing chromatograms of the participating individuals of family 2. Nucleotide and amino acid sequences are shown under the chromatograms. The nucleotide affected by the mutation is underlined and indicated with a red arrow (NM_001399.5: c.787A>C p.(Lys263Gln)). Individual identifications are indicated on the left side of each chromatogram. The changed amino acid is shown in red. (**F**) Homologene alignment. The lysine at the 263 codon position is completely conserved among vertebrates and indicated with a red arrow. The asterisk under the alignment indicates the conserved amino acid position (protein reference sequences are the same as in the Figure 1 legend). (**G**) Three-dimensional (3D) protein modeling image by the PyMOL program. The secreted EDA homotrimer form is shown. Each chain is shown in green. The lysine at the 263 codon position is shown as magenta spheres. The upper left is the top view, the upper right is the bottom view, and the lower left is a side view. The lower right image shows the EDA monomer interacting with EDAR (yellow).

The proband of family 2 was a three-year-old boy from a non-consanguineous family. Six deciduous teeth and eleven permanent teeth were missing (Figure 2). No other birth defects were associated with the nails, hair, skin, or sweat glands. His mother had microdontia in her lateral incisors, and the other immediate family members had no dental or systemic abnormalities. Therefore, we concluded that he presented features of NSO.

Mutational analysis of the proband revealed a novel missense mutation (c.787A>C p.(Lys263Gln)) in exon 6 of the *EDA* gene. The mother of the proband also carried the same mutation in the heterozygous form. However, the father did not have the mutation. The mutation was predicted to be pathogenic in silico (Table 2), and the amino acid was completely conserved among species. The 3D structure analysis revealed that residue Lys263 is located on the outer surface and in the bottom area of the homotrimer. This location interferes with signal transduction by binding its receptor, EDAR. In addition to the conformational change in the homotrimer itself, the electric charge of the side chain would be significantly changed from a positive charge to neutral by the p.(Lys263Gln) substitution.

## 4. Discussion

In this study, we identified one novel p.(Lys263Gln) mutation in the proband with NSO and a recurrent missense p.(Arg153Cys) mutation in the proband with X-linked ED. Few studies have reported the correlation between the phenotypes and genotypes of these two conditions [17,33]. Concerning the genotype, *EDA* mutations causing NSO mainly comprise missense mutations located in the TNF domain and retain a residual EDA receptor-binding activity. In comparison, the mutations responsible for X-linked ED are generally distributed across all of the EDA domains and might abolish the EDA signaling pathway [34]. The pattern of the *EDA* mutations in this study was consistent with previous results. Similarly, the phenotype pattern was similar to that reported in earlier studies, although it was not perfectly correlated. According to a report addressing the tooth agenesis pattern, the absence of incisors in both conditions did not differ significantly. However, for X-linked ED, the missing percentage of posterior teeth was higher than that of anterior teeth, and the remaining incisors generally presented an abnormal shape (peg shape). In comparison, the anterior teeth were more likely to be missing, whereas the molars were the least affected in NSO. Among the anterior teeth, the lower incisors and upper lateral incisors were most frequently missing [35]. Although the relationships between genotype and phenotype have not yet been established, this can help detect a potential mutation through the pattern of missing teeth, as in the present study [35].

**Figure 3:**
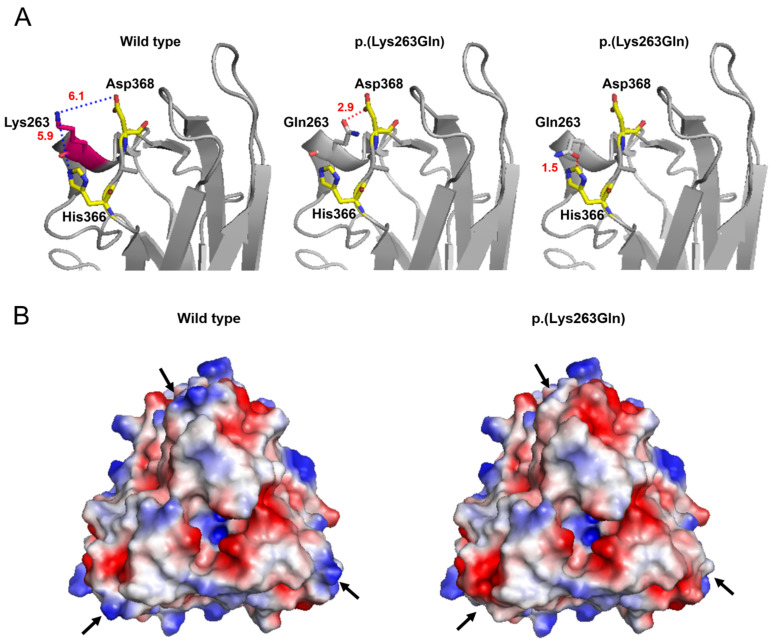
Three-dimensional (3D) protein modeling of the wild-type and the p.(Lys263Gln)-mutant proteins: (**A**) Relationships between Lys263 and neighboring residues. The side chain of Lys263 does not form any interactions within the monomer but maintains a certain distance from the neighboring residues in the wild-type protein. The most likely mutation-predictive models, with probabilities of 22.5% (middle) and 13.4% (right), are shown. (**B**) Electrostatic potential representation of the wild-type protein and p.(Lys263Gln)-mutant protein structures. Blue, white, and red represent the positive, neutral, and negative charges, respectively. The p.(Lys263Gln) mutation substantively alters the electropotential of the indicated region (black arrows). It can be seen that the positive charge of the surface is decreased overall in the mutant.

In this study, we detected a novel missense mutation (c.787A>C p.(Lys263Gln)) located in the TNF domain of EDA. This residue is an evolutionarily conserved amino acid, suggesting that Lys263 may play a crucial role in the function of the EDA protein [36]. The mutation may have affected the stability of the protein itself or the homotrimer [37]. According to the analysis of the 3D protein model, the side chain of Lys263 does not form any interactions within the monomer, instead maintaining a certain distance from the adjacent His366 and Asp368 residues (Figure 3A). Substitution of Lys with Gln would reduce the distance between these residues and result in the rearrangement of their positioning or the deformation of the local structure. In turn, these structural changes might compromise the stability of the monomer or the homotrimer. This substitution would also change the protein surface charge, and this change in electric charge may partially interfere with the interaction with EDAR (Figure 3B) [37,38,39].

In addition, our study also confirmed a known p.(Arg153Cys) mutation in the proband of family 1 with X-linked ED. This mutation has been reported in previous studies [2,23,35]. It is located in the furin protease recognition sequence, which is necessary for the proteolytic cleavage of EDA. The p.(Arg153Cys) mutation has been shown to disrupt one of two furin recognition sites. Still, the Cys appears to form a novel disulfide bridge, distorting the local structure of the EDA around the furin active site. As a result, the mutation can disrupt the release of soluble EDA, leading to the development of relatively milder phenotypes than the p.(Arg156Cys) mutation, which completely abolishes the EDA processing. The phenotype of the proband of family 1 appeared to be consistent with these previous findings.

This study successfully identified two EDA missense mutations associated with oligodontia exhibiting variable expression of ED, one of which is novel. The potential disease-causing mechanisms were elucidated through Western blot analysis and structural modeling. However, we must acknowledge a limitation in our approach: direct signaling effects, such as those that could be demonstrated through luciferase assays, could not be investigated due to technical constraints in our laboratory. This represents an opportunity for future research to further validate and expand upon our findings.

## 5. Conclusions

In summary, we identified one novel missense mutation and one reported mutation in the *EDA* gene in two oligodontia families. Our findings suggest that these mutations could cause tooth agenesis by impairing the EDA protein structure and processing. The findings of this study may contribute to a better understanding of the molecular mechanism of the EDA signaling pathway. Moreover, further functional analysis of the mutations may provide insights into the mechanisms of tooth agenesis and variable expressivity.

## Figures and Tables

**Table 1:** Mutagenesis primers.

	Forward Primer	Reverse Primer
c.457C>T p.(Arg153Cys)	5′-GAAGAAAGTAGGTGTGTTCGCCGC-3′	5′-GCGGCGAACACACCTACTTTCTTC-3′
c.787A>C p.(Lys263Gln)	5′-GCAATTCAAGTCCAGAATGATCTTTC-3′	5′-GAAAGATCATTCTGGACTTGAATTGC-3′

**Table 2:** *EDA* mutations identified in this study.

Patient ID	*EDA* Mutations	Missing Teeth (FDI Notation)	Diagnosis
II:1 family 1	c.457C>T p.(Arg153Cys)	#52, 62, 71, 72, 73, 81, 82, 83 12, 13, (15), (17), 22, 23, (25), (27), 31, 32, 33, (34), (35), (37), 41, 42, 43, (44), (45), (47)	X-linked ED
III:2 family 4	c.787A>C p.(Lys263Gln)	#52, 62, 71, 72, 81, 82 12, 15, 22, 25, 27, 31, 32, 35, 41, 42, 45	Oligodontia

The tooth numbers in parentheses indicate that the patient was too young to confirm these missing teeth.

**Table 3:** In silico prediction of the mutations.

	PolyPhen-2	Mutation Taster	CADD 1.7
c.457C>T p.(Arg153Cys)	Possibly damaging (score: 0.853)	Disease-causing (prob: 0.889)	23.1
c.787A>C p.(Lys263Gln)	Probably damaging (score: 0.992)	Disease-causing (prob: 0.994)	41

## Data Availability

The data presented in this study are openly available at ClinVar (http://www.ncbi.nlm.nih.gov/clinvar/, accessed on 26 July 2024), Accession ID: SCV005088518.
